# Gene therapy for cancer: present status and future perspective

**DOI:** 10.1186/2052-8426-2-27

**Published:** 2014-09-10

**Authors:** Magid H Amer

**Affiliations:** Department of Medicine, St Rita’s Medical Center, 825 West Market Street, Suite #203, Lima, OH 45805 USA

**Keywords:** Adenoviruses, Clinical trials, Electroporation, Gene silencing, Gene transfer technique, Immunomodulation, Molecular targeted therapy, Oncolytic viruses, Retroviruses, Suicide transgenes

## Abstract

Advancements in human genomics over the last two decades have shown that cancer is mediated by somatic aberration in the host genome. This discovery has incited enthusiasm among cancer researchers; many now use therapeutic approaches in genetic manipulation to improve cancer regression and find a potential cure for the disease. Such gene therapy includes transferring genetic material into a host cell through viral (or bacterial) and non-viral vectors, immunomodulation of tumor cells or the host immune system, and manipulation of the tumor microenvironment, to reduce tumor vasculature or to increase tumor antigenicity for better recognition by the host immune system. Overall, modest success has been achieved with relatively minimal side effects. Previous approaches to cancer treatment, such as retrovirus integration into the host genome with the risk of mutagenesis and second malignancies, immunogenicity against the virus and/or tumor, and resistance to treatment with disease relapse, have markedly decreased with the new generation of viral and non-viral vectors. Several tumor-specific antibodies and genetically modified immune cells and vaccines have been developed, yet few are presently commercially available, while many others are still ongoing in clinical trials. It is anticipated that gene therapy will play an important role in future cancer therapy as part of a multimodality treatment, in combination with, or following other forms of cancer therapy, such as surgery, radiation and chemotherapy. The type and mode of gene therapy will be determined based on an individual’s genomic constituents, as well as his or her tumor specifics, genetics, and host immune status, to design a multimodality treatment that is unique to each individual’s specific needs.

## Introduction

Over the last two decades, cancer research and genomics have experienced considerable advancements. In 2001, two independent draft versions of the human genome sequence and the concomitant identification of approximately 30,000 genes were published [1, 2]. As expected, this was followed by extensive research in molecular genetics with advanced tools used to dissect gene function and explore the biological processes involved in causing genetic aberration and malignant transformation [3]. The recent proliferation of knowledge of cancer has led to the development of novel therapeutic approaches in cancer management, particularly gene therapy.

Gene therapy implies any procedure intended to treat or alleviate a disease by genetically modifying the cell of a patient [4]. The material to be transferred into patient cells may be genes, gene segments, or oligonucleotides. Gene transfer therapy can be conducted either as in vivo or ex vivo approaches. In the in vivo approach, targeted cells are approached directly, such as the intradermal injection of a metastatic nodule, or intravesical therapy for superficial bladder cancer. In the ex vivo approach, targeted cells from a tumor are selected, then collected, grown in culture media at a controlled microenvironment, manipulated genetically by the insertion of a new gene or protein (transgene) in the cell genome, then introduced back into the host. The ex vivo approach is much simpler to achieve as it is easier to manipulate target cells externally.

Concerning cancer, initial efforts to deactivate oncogenes and replace non-functioning tumor suppressor genes were barely successful. Subsequently, new approaches have been developed to transfer genetic materials (transgenes) directly into target cells aiming to transiently or permanently change their phenotypes [5]. Target cells may be normal cells, cancerous cells, immune mediated cells, or pluripotent stem cells. Once the transgene enters a cancer cell, it can then assists in its death or restore normal cellular functions, whereas for normal cells, the transgene can protect them from drug-induced toxicities, or activate an immune cell to get rid of the cancer cell. Gene and vector-based molecular therapies for cancer comprise a wide range of treatment modalities to modify cancer cells, normal cells, and/or a tumor microenvironment [6].

## History

The history of cancer therapy dates back to the eighteenth century, when surgery was the primary treatment for early stages of cancer, and patients suffered from frequent relapses [7]. Once the disease spread, patients were treated with herbal medications, castor oil, or arsenic. In 1895, radiation therapy was discovered, but resulted in few cures [8]. At that time, several cases of spontaneous cancer regression following bacterial infection were reported [9]. In 1868 a patient with soft tissue sarcoma went into remission following an erysipelas infection, but this regression lasted for a short duration [9]. In 1943, nitrogen mustard was used in the management of patients with lymphoma [10], and in 1948 folic acid antagonists led to transient remission in childhood leukemia [11]. Since then, there has been a dramatic advancement in chemotherapy treatment for cancer [7]. Viruses were also found to be effective in controlling malignancies in animal models, and subsequently in humans in 1956 [12]. Adenoviruses in particular have been studied more intensively in humans, with the subsequent development of gene therapy [13]. In 1987, immunotherapy was introduced in the management of cancer patients with subsequent FDA approval of rituximab antibodies in the treatment of patients with lymphoma (1997) [14].

The first FDA-approved gene therapy experiment in the United States occurred in 1990 for a patient with severe combined immunodeficiency disorder [15]. Since then, many clinical trials have been conducted for patients with cancer, using different approaches in gene therapy, with successful results reported in patients with chronic lymphocytic leukemia, acute lymphocytic leukemia, brain tumors, as well as others. Several commercially approved medications for gene therapy were released, including ONYX-15 (Onyx Pharmaceuticals) for refractory head and neck cancer (2005) [16]; human papilloma virus vaccine (Gardasil) (Merck Sharp & Dohme) for the prevention of cancer cervix (2006) [17]; and modified dendritic cells, sipuleucel-T (Provenge) (Dendreon Corporation, Seattle, WA), for minimally symptomatic, castration-resistant metastatic prostate cancer (2010) [18].

## Methods of gene therapy

Gene therapy implies an approach that aims to modify, delete, or replace abnormal gene(s) at a target cell. Such target cells may be malignant primary or metastatic nodules, circulating tumor cells or dormant stem cells, and specific cells such as T-cell lymphocytes or dendritic cells. With the presence of over 20,000 active genes in human cells, exposed to numerous factors whether hereditary, environmental, infectious or spontaneous, unlimited possibilities for gene mutation, aberration, dysfunction or deletion have been expected, leading to clinical presentation of various medical disorders, including cancer. Furthermore, genomics of cancer evolve between primary and metastases. For example, estrogen receptor gene (ESR J) mutations in breast cancer were found in proportion of metastases but not in primary tumors [19, 20]. Whole-exome sequencing of metastatic samples reported among the top 17 mutated genes, only five were mutated in primary tumors [21]. The evolution from minority clone to lethal metastases follows branched evolution. Thus, tumors with high a level of intratumor heterogenicity and genomic instability could be more likely to escape from targeted therapies such as gene therapy, unless such a branched evolution is taken into consideration. Hence, gene therapy is somewhat difficult to achieve, with limited success. Presently, most approaches are for monogenic gene therapy, tackling one or more critical gene defects. Selection of the appropriate mode of gene therapy is based on the assessment of the immune status, and determination of the molecular nature of a patient’s disease. With the recent increases in knowledge of molecular biology of various medical disorders, a more advanced and comprehensive gene therapy approach will ultimately become available, with anticipated improved results.

## Gene transfer delivery system

Several methods have been developed to facilitate the entry of genetic materials (transgenes) into target cells, using various vectors. They are broadly divided into two major categories: viral (or bacterial) and non-viral vectors [Table 1]. Viruses usually bind to target cells and introduce their genetic materials into the host cell as part of their replication process. As they enter target cells, they can carry a load of other genetic material called “transgenes”. For non-viral vectors, different approaches have been utilized, using physical, chemical, as well as other modes of genetic transfer. Transferring genetic material directly into cells is referred to as “transfection”, while moving them into cells carried by a viral or bacterial vector is termed “transduction”. Non-viral approaches have the advantage of safety and easy modifiability, but have a lower transfection efficiency compared to viral vectors [22].

**Table 1:** **Gene transfer and immunomodulation in cancer therapy**

Predominant action	Examples	Commercially available*	Clinical trials, Phases II,III,IV **
**Gene transfer**			
Non-Viral	Electroporation, nanoparticles, hydrodynamics, cationic liposomes, transposon, synthetic viruses		18,1,0
Bacterial	Escherichia coli, Salmonella, Clostridium, Listeria, CEQ508		6,0,0
Viruses			
ssDNA viruses	Adeno-Associated: Parvovirus		
dsDNA viruses	Adenoviruses: Ad5-D24, CG870, Ad5-CD/TKrep, Recombinant H103, Gutless adenovirus, OBP-301	ONYX-015	11,3,0
dsDNA viruses	Herpetic viruses: Herpes simplex-1, TVEC		42,10,0
ssRNA viruses	Lentiviruses: HIV-1, HIV-2, Simian IV, Feline IV.		8,2,0
dsRNA viruses	Reoviruses		9,1,0
**Immunomodulation**			
Active immunotherapy			41,3,0
	Single Tumor cell surface antigen vaccine		
	Antigen-specific plasmid-based vaccine: PSA, HER/2, Modified CEA vaccine.		
	Tumor cells, irradiated as vaccine		
	Genetically modified tumor cell vaccine: Using Poxvirus, Vaccinia virus, Recombinant fowlpox virus, Combination (TRICOM) (Prostvac-VF vaccine).		
Passive immunotherapy			219,29,2
	Antibodies against:	Rituximab	
	CD20 Protein on lymphoma cells	Rituximab	
	HER/2 receptor protein in breast cancer	Trastuzumab	
	CD52 Protein on CLL	Alemtuzumab	
	CD20 Protein on lymphoma cells	Ibritumomab	
	CD20 Protein on lymphoma cells	Tositumomab	
	EGFR Receptor on squamous CA	Cetuximab	
	EGFR Receptor on colorectal CA	Panitumumab	
	CD20 Protein on CLL	Ofatumumab	
	CD30 Protein on Hodgkin lymphoma cells	Brentuximab	
	HER/2 receptor protein in breast cancer	Pertuzumab	
	HER/2 receptor protein in breast cancer	Ado-Trastuzumab	
	CD20 Protein on CLL	Obinutzumab	
Adoptive immunotherapy			15,1,0
	Autologous activated T- lymphocytes	Sipuleucel-T	
	Genetically modified activated T-lymphocytes		
	Chimeric antigen receptor integrated T-lymphocytes		
	Activated dendritic cells		
	Genetically modified dendritic cells		
Immune enhancement			11,1,0
	Antibodies blocking CTLA-4 Inhibitors for malignant melanoma.	Ipilimumab	
**Microenvironment modification**			
Impact on vasculature	Humanized monoclonal antibodies against VEGFR-A	Bevacizumab	
	Anti-angiogenic genes (against VEGFR-A): Endostatin, Angiostatin		22,4,0

*Abbreviations*: *CA* Cancer; *CEA* carcinoembryonic antigen; *CLL* chronic lymphocytic leukemia; *ds* double stranded; *CTLA-4* cytostatic T-lymphocyte antigen 4; *DNA* deoxy nucleic acid; *EGFR* epidermal growth factor receptor; *FDA* Food and Drug Administration in United States; *HER/2* human epidermal growth factor receptor-2; *HIV* human immunodeficiency virus; *PSA* prostatic acid phosphatase antigen; *RNA* ribonucleic acid; *ss* single stranded; *VEGF-A* vascular endothelial growth factor A receptor.

*Commercially approved medications by FDA US as of July 1, 2014. ONYX-015 was previously approved by FDA China.

**Clinical trials: Number of active clinical trials on gene therapy for cancer (Phases-II, -III, and –IV) as of July 1, 2014 (http://www.clinicaltrials.gov).

### Physical mediated gene transfer

DNA genetic material that is coated with nanoparticles from gold or other minerals, and with their kinetic energy supplemented by compressed air or fluid (gene gun), or using ultrasound, can force the genetic material into the target cell, followed by the release of DNA into its nucleus. They are best suited for gene delivery into tissue or in case of gene vaccination [23].

The electroporation gene therapy approach aims to achieve cellular membrane disruption with high-voltage electrical pulses, resulting in the formation of nanopores through which naked DNA, foreign genetic materials, and even chemotherapeutic agents can enter cells [23, 24]. This approach is best suited for plasmid DNA-based gene transfer therapy with the advantage of effectiveness in a vast array of cell types, ease of its administration, lack of genome integration with the risk of malignancy, as well as the low potential for unwanted immunogenicity [22]. Electroporation is presently being tested in several clinical trials, especially on patients with malignant melanoma, prostate cancer, colorectal cancer, and leukemia [22].

### Chemical mediated gene transfer

Cationic liposomes are microscopic vesicles of synthetic phospholipids and cholesterol that can enter into cells by endocytosis [25], with the capability of carrying a variety of molecules such as drugs, nucleotides, proteins, plasmids and large genes [23]. Their advantage is selectivity to endothelial cells, a relatively high rate of gene transfer efficiency, a broad application as carriers for many genes, and the lack of severe side effects [26]. When combined with small interfering RNA (siRNA), cationic liposomes may lead to the inhibition of tumor proliferation, inducement of apoptosis, and enhancement of radiosensitivity to tumor cells [27].

Synthetic viruses have been developed to exploit the efficiency of viral vectors and the advantage of liposomes [28]. Once they enter the target cell, DNA is released from the endosome. This method has shown promising results in preclinical studies [29–32]. Transposons can also transport genetic material inside the cell as well as into the nucleus [33].

### Bacterial mediated gene transfer

Some bacteria have the capability of specifically targeting tumor cells, leading to RNA interference (RNAi) and gene silencing with blockage of RNA functions, including cellular metabolism and protein synthesis. Examples include Escherichia coli, Salmonella typhimurium, Clostridium, and Listeria [34]. Bacterial vectors can deliver pro-drug-converting enzymes and cytotoxic agents into tumor cells, and can mediate the host immune response. They can be engineered to carry magnetic or fluorescent material to enhance the utility of diagnostic approaches in tumor localization, such as with magnetic resonance imaging (MRI) [35], and even in the development of cancer vaccines [36]. However, the outcome has been far less pronounced compared to other RNA interference silencing techniques. Overall, genetically engineered bacteria acting as vectors for RNA interference are relatively safe, effective, practical and cheaper to manufacture compared to viral vectors. They selectively colonize and grow within the tumor. They can also be administered orally, hence their use in the management of gastrointestinal disorders [34].

### Viral mediated gene transfer

Viruses are small particles that contain either ribonucleic acid (RNA) or deoxyribonucleic acid (DNA), and may be single-stranded (ss) or double-stranded (ds). The viral structure consists of a genome surrounded by a protective protein coat (viral capsid) which helps the virus attach to host cell receptors, and prevents viral destruction by cell nuclease enzymes. Some viruses may also have a lipid bilayer envelope derived from the host cell’s membrane, and an outer layer of viral envelope made of glycoprotein. A complete viral particle (virion) by itself is unable to replicate. For propagation, the virus needs to insert its genetic material into a host cell, in order to acquire metabolic and biosynthetic products for viral transcription and replication.

Viruses can be modified genetically to be noninfectious. As they enter the cell, they can carry genetic material for delivery into a target cell’s cytoplasm, and subsequently into the nucleus. In monogenic gene therapy, virus vectors can carry a load of 2–10 kb of relevant genes. In high complex gene therapy, other supporting molecules can also be added, such as immune-stimulatory molecules to the virus’s DNA for subsequent release during viral replication. The advantage of viral vectors in gene therapy is the ease of purification into high titers, and prolonged gene expression with minimal side effects. Retroviruses including lentiviruses can integrate themselves into host cell genome at the nucleus, while adenoviruses and adeno-associated viruses predominantly persist as extrachromosomal episomes [24, 37].

RNA viruses comprise about 70% of all viruses, and vary greatly in genomic structures. They usually have a higher mutation rate with increased adaptation to attack different host cells. Single-stranded RNA viruses may have a viral reverse transcriptase enzyme in their genome, which helps in genetic transcription of the viral genome inside the host nucleus, into double-stranded pro-viral DNA. With viral integrase, the pro-viral DNA then integrates with the host DNA making the subsequent transcription of other parts of the virus possible, in order to give rise to a new retrovirus progeny. The proteins of the mature virion are then rearranged to form the new viral particles. Viral particles subsequently destroy the host cell, and release mature viruses to attack neighboring cells. Double-stranded DNA viruses enter the host cells by endocytosis through interaction between the virus and cell receptor. The virus then enters the nucleus through nuclear pores once they escape cellular endosome [38, 39]. The virus subsequently releases two gene products which bind to the retinoblastoma and p53 tumor suppressor genes, thus allowing viral replication. New viruses then cause cell lysis and the released viruses spread to attack neighboring cells [38].

#### Adenoviruses

Adenoviruses are double-stranded DNA viruses that usually cause mild respiratory, digestive and ocular infection in humans. In gene therapy, modified versions of adenovirus and adeno-associated viral vectors have been designed. Compared to wild-type, they are more potent in infecting cells, both dividing and non-dividing, replicate exclusively in tumor cells [40], and selectively target certain cellular receptors or molecular defects. They pose a very high transduction efficiency, which may approach 100%, with fewer tendencies for viral shedding and latent infection. They can easily be produced commercially in large quantities, and are capable of carrying pro-drug genes as well as others [41]. However, they have several pitfalls, including the tendency to develop genetic instability of carried genes. Subsequent chromosomal aberration may lead to the development of lymphoproliferative disorders. As nearly half of all humans have been exposed to these viruses during their lifetime, with the generation of neutralizing antibodies, they may lead to high immunogenicity, with shorter duration of adenoviral-mediated transgene expression [42].

Modified oncolytic adenoviruses are presently tested in different clinical trials, especially in patients with astrocytoma of the brain, in combination with radiation and/or temozolomide chemotherapy [43]. ONYX-015 (Onyx Pharmaceuticals) is a modified oncolytic adenovirus that was previously approved by the Chinese Food and Drug Administration (2005) in the management of refractory head and neck cancer in combination with cisplatinum [16]. It is presently being investigated in the management of other solid malignancies. Other oncolytic adenoviruses include Ad5-D24, recombinant H103, Ad5-CD/TKrep, CG7870, KH901, and OBP-301 (Telomelysin). The latest generation of adenoviral vectors is the gutless adenovirus; it has an impressive safety profile, less in vivo immune response and long-term sustained gene expression [24]. Most clinical trials using oncolytic adenoviruses rarely produce dramatic tumor response. However, when combined with other modalities of cancer therapy, good tumor regression has been reported [38, 42].

#### Adeno-associated virus

This represents small, single-stranded DNA viruses, which do not usually cause infection without co-infection of a helper virus, such as adenovirus, or herpes simplex virus. They have the advantage of broad host range, low level of immune response, and longer gene expression. One example is the Eukaryotic adeno-associated virus, which is a chimeric virus vector containing parvovirus and adenovirus [44]. It is capable of transfecting mitotic and quiescent cells, lacks immunogenicity and pathogenicity in humans, and integrates stably into the host DNA at a predictable location within a chromosome-19 in cell culture, but not in mammalian cells.

#### Herpes simplex virus

This is a large, enveloped double-stranded DNA virus (150 kb), naturally neurotropic (prefer nerve cells), that infects humans particularly at the oral and genital mucosa, but ultimately spreads to sensory nerves to replicate or become dormant at the sensory ganglions. Viral reactivation may lead to oral or genital ulcerations, skin rashes, or even encephalitis. Up to 80% of the population are seropositive to the virus [45, 46]. With genetic engineering, a modified oncolytic recombinant replication-selective herpes simplex virus has been developed, and has exhibited several advantages: it has broad tropism, potent in causing tumor cell lysis, it is non-integrating in targeting the cell genome (apart from nonessential genes), can evade the host immune system; and in case of toxicities, several effective antiviral therapies are presently available to control viral replication. Another advantage is its viral capability to carry a large load of transgenes, such as a pro-drug-activating gene thymidine kinase enzyme that enhances tumor lysis when ganciclovir medication is subsequently administered intravenously (suicide gene) [45]; therapeutic immunomodulatory transgenes that augment the antitumor immune response (such as talimogene laherparepvec) (OncoVEX GM-CSF) [47]; and antiangiogenic genes to suppress tumor vasculature [48]. Presently, modified oncolytic herpes simplex viruses such as Talimogene laherparepvec (TVEC) as well as others, are being tested in several clinical trials either as a monotherapy, or in association with surgery, radiation therapy or chemotherapy, particularly on patients with high-grade glioma. Currently, some success has been reported [45].

#### Lentivirus vector

Lentiviruses are retroviruses that infect bovine, equine, nonhuman primates and humans [49]. One of the most destructive human pathogens is human immunodeficiency virus infection (HIV). It constitutes a class of enveloped viruses that contain a single-stranded 9.2 kb RNA genome. The lentivirus carries a reverse transcriptase enzyme that transcribes RNA into double-stranded DNA once it enters the cytoplasm. It then integrates permanently into the nuclear genome of the target cells. Examples include lentiviral vectors derived from immunodeficiency viruses such as HIV-1, HIV-2. With genetic engineering, researchers have removed the infectious parts of the virus and added other parts from different viruses such as cytomegalovirus, generating a highly modified lentivirus [50]. Another genetic modification employed by previous researches created an integration-deficient lentiviruses that did not integrate into a host genome [51, 52], though with slightly lower transduction efficiency. Such a modified lentivirus has the advantage of being relatively safe, of having variable specificity to either a particular cell or is broad enough to infect all cells, and of having efficient transduction of both dividing and non-dividing cells. Modified viruses have low antivirus immunity, low potential for genotoxicity due to insertional mutagenesis, and the capability of carrying genes inside the nucleus [49]. Major disadvantages include inadequate immune responses as well as antitumor response, the risk of viral transformation into pathogenic HIV infection, especially in immunized individuals, and insertional mutagenesis of new cancer genes into the host genome, with the risk of second malignancy [49].

#### Reovirus

This is an oncolytic virus that usually infects animals. In humans, it rarely causes major illness except for respiratory and gastrointestinal symptoms. Nearly 100% of human adults are seropositive to the virus [53]. It is non-enveloped, double-stranded RNA (dsRNA), and its oncolytic activities are mainly through stimulation of the immune system, particularly through bystander immune activation. The release of tumor-associated antigens following cellular lysis further stimulates innate immunity against tumor cells. The virus is considered relatively benign, with good safety records, does not require genetic alterations to become an oncolytic virus, and is less expensive to be produced commercially. Because of its relative safety, the virus is presently being used in several clinical trials, as oncolytic reovirus monotherapy, administered intratumoral, intravenously, or intraperitoneally; or as polytherapy, in combination with radiation therapy or chemotherapy [53].

## Gene therapy implementation

Once genetic materials are transferred into target cells and incorporated into nuclear genetic DNA, they may induce silencing, down-regulation, modification, or repair of the target cell genes. Depending on the intensity of the gene expression, it may lead to cell death and tumor necrosis (as with the suicide gene), or impaired cell growth with tumor regression (as with the silencing gene). Modification of the gene may improve the response from subsequent cancer therapy, such as chemotherapy, immunotherapy, or radiation. Repair of the target gene may help in preventing subsequent malignancy or cancer-related complications such as thrombosis. They may also be helpful in the future by preventing hereditary cancer syndromes.

### Suicide gene

**Figure 1:**
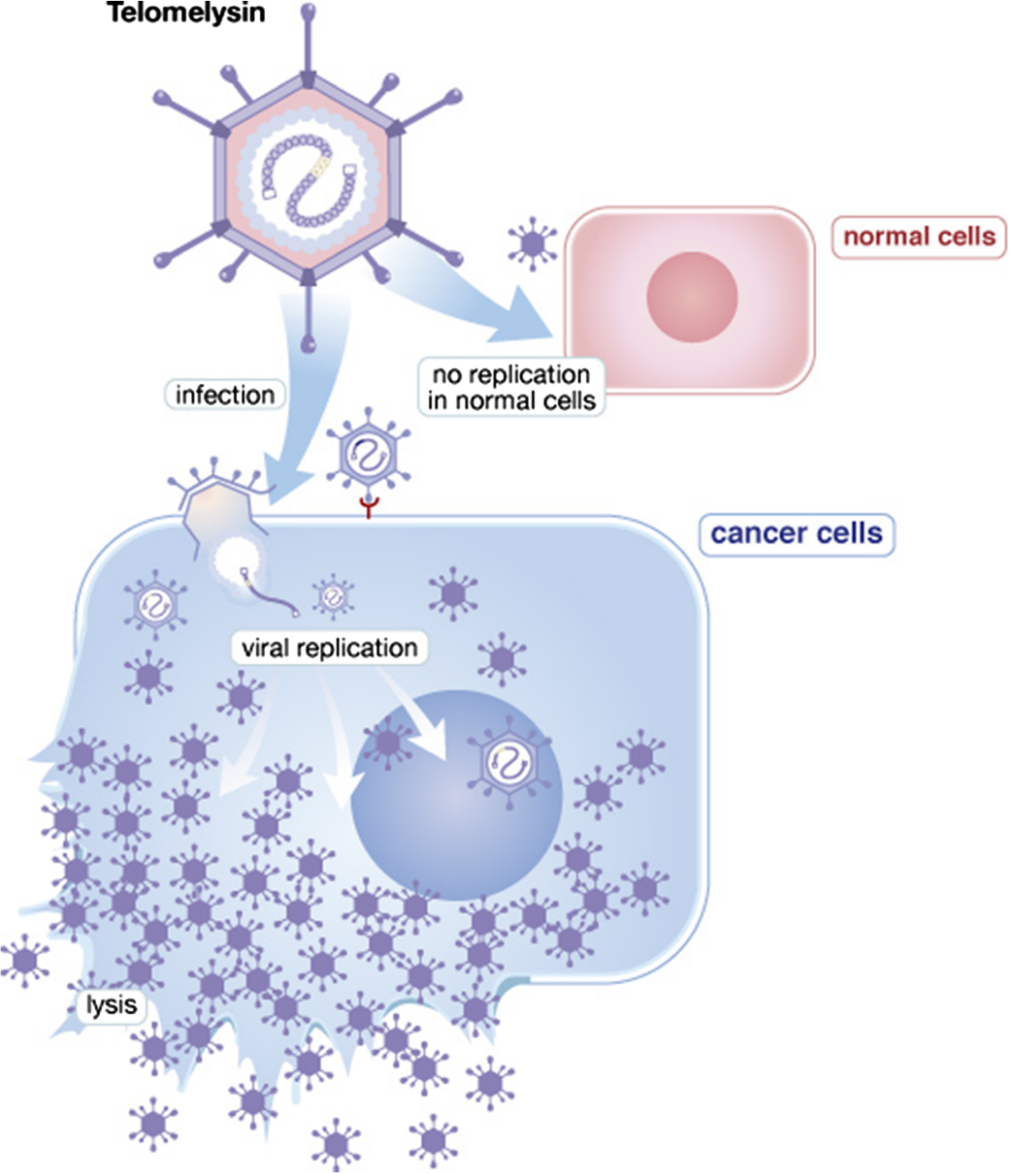
**Genetically-modified adenovirus acting as a suicide gene.** The above mode of action represents an example of a modified virus acting as a suicide gene, namely OBP-301 (Telomelysin) (Courtesy Oncolys BioPharma Company, Tokyo, Japan).

These are transgenes that make up products that can cause a cell to kill itself through apoptosis. Such gene products are usually transcribed by various factors (promoters) leading to cell death and necrosis. One example of such promoters is the human *H19* RNA gene which is highly expressed in most fetal organs but rapidly cleared immediately after birth [54]. This gene has shown an abnormal expression in various types of cancer cells, and plays an important role in cancer cell proliferation, genetic instability, vascular angiogenesis, tumor metastases, multidrug resistance as well as cell survival despite hypoxia, with secondary tumor progression and dissemination [55]. Blocking *H19* gene function leads to marked tumor regression, cellular death and necrosis. Another important promoter is human telomerase reverse transcriptase (hTERT), which is a critical factor for cell immortalization and tumorigenesis [56]. Its blockage with agents such as OBP-301 (Telomelysin) (Oncolys BioPharma) leads to cell necrosis and tumor regression (Figure 1). Other approaches to induce tumor cell death include the use of small-molecule drugs, monoclonal antibodies, and toxin gene therapy with agents such as Corynebacterium diphtheriae toxin-A chain (DTA-H19 therapy) [57].

### Gene silencing

This has been achieved through specific delivery of a small interfering double-stranded RNA (siRNA) into target cells, and subsequent duplex formation of RNA-induced silencing complex (RISC) that destroys messenger-RNA (mRNA), thus leading to interference with RNA functions and protein synthesis within the target cells [58]. Through the appropriate design of siRNA, it is theoretically possible to use the technology in silencing any gene in the body, providing a greater therapeutic potential in cancer therapy [59], as well as in the management of other medical disorders such as the hepatitis B virus, human papilloma virus, hypercholesterolemia and liver cirrhosis [59, 60]. As siRNA does not interact with chromosomal DNA, it does have a lower risk of inducing target cell gene alterations and possible mutagenesis. It is highly specific against target genes, with low systemic toxicities, and does not induce multidrug resistance. Furthermore, these genes can induce potent gene silencing of many cancer-related genes, leading to tumor regression, but do not abolish abnormal genes. siRNA therapy can be administered directly into tumors; however, for systemic administration, it is somewhat difficult as a naked siRNA protein is liable for host-mediated clearance by enzymatic degradation, renal filtration, and host cellular phagocytosis. Several delivery systems for siRNA have been developed to protect them from enzymatic degradation, and facilitate their effect in silencing specific genes. Examples of siRNA systemic delivery system presently in clinical trials include CALAA-01 (Calando Pharmaceuticals) for patients with malignant melanoma [61], and ALN-VSPOI (Alnylam Pharmaceuticals) for liver cancer and solid tumors [62]. However, limited success has been achieved mainly due to relatively high toxicity and low transfection efficiency [58, 59].

### Gene modification

This may be helpful in improving cancer therapy results, such as with radiation therapy. Radiosensitizing gene therapy promotes transgene expression in tumor tissue, thus increasing tumor sensitivity to radiation with better tumor control [63–65]. In contrast, radioprotective gene therapy distributes transgenes and their products to surrounding normal tissue, thus limiting radiation induced toxicities to normal tissue [66]. The concept of combining both approaches is presently being investigated in several preclinical studies.

### Gene repair

This can be achieved using zinc finger nuclease attached to the lentiviral vector. Once the viral vector enters the nucleus, it binds to a specific location in the double-stranded DNA, breaking it at specific location, with subsequent endogenous repair mechanisms, to create a newly edited double-stranded DNA [23]. Other technological approaches include transcription activator-like effector nucleases (TALENs) [67, 68], and clustered regularly interspaced short palindromic repeats (CRISPR) [69].

### Gene therapy for mitochondria

Gene therapy may also be directed to cytoplasmic organelles such as mitochondria. The mammalian mitochondria are responsible for metabolic functions. Nearly 300 of the known mutations causing metabolic diseases are secondary to disorders affecting the mitochondrial genome [23]. Several approaches have been used to transfer genes successfully into cell mitochondria.

## Immunomodulation

It has become apparent that the immune system is a crucial element in cancer regression or progression. There are two types of immune responses: humoral immunity and cellular immunity. Furthermore, the tumor microenvironment plays an important role in host immune effects against cancer cells [Table 1].

Humoral immunity is mediated by antibodies released by B-cells with a high-binding affinity to specific tumor antigens. The Food and Drug Administration in the United States (FDA) approved several antibodies against malignant cells, which include trastuzumab for breast cancer [70], rituximab for indolent lymphoma [71], cetuximab for lung cancer [72], and bevacizumab for various solid tumors [49, 73], and many others [74] [Table 2].

**Table 2:** **Monoclonal antibodies in cancer management**

Name	Class	Target	Approved initial indications	FDA Approved
Rituximab (Rituxan)	Chimeric IgG1	CD20	Non-Hodgkin lymphoma	1997
Trastuzumab (Herceptin)	Humanized IgG1	HER2	Breast cancer	1998
Alemtuzumab (Campath)	Humanized IgG1	CD52	B-cell CLL	2001
Ibritumomab tiuxetan (Zevalin)	Murine IgG1	CD20	Non-Hodgkin lymphoma	2002
Tositumomab (Bexxar)	Murine IgG2a	CD20	Non-Hodgkin lymphoma	2003
Cetuximab (Erbitux)	Chimeric IgG1	EGFR	Squamous cancer head & neck	2004
Bevacizumab (Avastin)	Humanized IgG1	VEGF	Colorectal cancer	2004
Panitumumab (Vectibix)	Humanized IgG2	EGFR	Colorectal cancer	2006
Ofatumumab (Arzerra)	Humanized IgG1	CD20	CLL	2009
Denosumab (Xgeva)	Humanized IgG2	RANKL	Bone metastases	2010
Ipilimumab (Yervoy)	Humanized IgG1	CTLA-4	Metastatic melanoma	2011
Brentuximab vedotin (Adcetris)	Chimeric IgG1	CD30	Hodgkin lymphoma	2011
Pertuzumab (Perjeta)	Humanized IgG1	HER2	Breast cancer	2012
Trastuzumab emtansine (Kadcyla)	Humanized IgG1	HER2	Breast cancer	2013
Obinutzumab (Gazyva)	Humanized IgG1	CD20	B-cell CLL	2013
**Name**	**Class**	**Target**	**Present indications**	Clinical Trials**
Amatuximab	Chimeric IgG1	mesothelin	mesothelioma	Phase-I
Elotuzumab	Humanized IgG1	CS1	Multiple myeloma	Phase-III
Farletuzumab	Humanized IgG1	FRA	Ovarian and lung cancers	Phase-III
Inotuzumab ozogamicin	Humanized IgG4	CD22	ALL, Malignant lymphoma	D/C
Moxetumomab pasudotox	Murine Fv-CD22	CD22	Hairy cell Leukemia	Phase-III
Naptumomab estafenatox	Murine Fab	5 T4	Renal and solid malignancies	Phase-II
Necitumumab	Humanized IgG1	EGFR	NSCL (Squamous cell)	Phase-III
Nivolumab	Humanized IgG4	PDI	NSCL, Melanoma, Renal	Phase-III
Ontuximab	Humanized IgG1	TEM1	Solid tumors	Phase-I/II
Onartuzumab	Humanized IgG1	c-Met	NSCL, Gastric	D/C
Racotumomab vaccine (Vaxira)	Murine	GM3	NSCL	Phase-III
Rilotumumab	Humanized IgG2	HGF/SF	Gastric, GEJ	Phase-III

*Abbreviations*: *5 T4* Antigen expressed on several solid tumors; *ALL* acute lymphocytic leukemia; *c-MET* MNNG HOS proto-oncogene that encodes hepatocyte growth factor receptor; *CLL* chronic lymphocytic leukemia; *CTLA-4* cytotoxic T lymphocyte inhibitors mediated by malignant cells; *CS1* human CS1 antigen glycoprotein belonging to CD2 subset of the immunoglobulin superfamily; *D/C* clinical trials that were discontinued due to the lack of efficacy or excessive toxicities; EGFR, epidermal growth factor receptor; *FDA* Food and Drug Administration in United States; *FRA* folate receptor alpha; *GM3* tumor antigen N-glycolil, a type of ganglioside present on the surface of breast and lung cancer cells; *HGF* human hepatocyte growth factor receptors; *Mesothelin* mesothelin is cell surface glycoprotein overexpressed in multiple malignancies such as mesothelioma; *NSCL* non-small cell lung cancer; *PD-1* human cell surface receptor programed death-1, results in activation of T cell mediated immune responses; *RANKL* RANK ligand protein that acts as the primary signal for bone removal, loss, or destruction; *TEM1* tumor endothelial Marker-1 and CD248 (Morphotek Inc, Exton, PA); *VEGF* vascular endothelial growth factor receptor.

**Active clinical trials on monoclonal antibodies in cancer management as of July 1st, 2014 (http://www.clinicaltrials.gov).

Cellular immunity is mediated by cell-to-cell contact that leads to antigen recognition and cell destruction of a target cell. Based on the intensity of tumor-associated antigens (TAAs) on the surface of tumor cells, they are recognized by the host immune system [75]. Dendritic cells are specialized in antigen recognition as well as mediation of immune responses against infectious agents or malignant cells, through direct stimulation or inhibition of immune effector cells such as T-cells, B-cells, and natural killer (NK) cells [76]. Dendritic cells are derived from the bone marrow and migrate to lymph nodes and distant tissue, looking for those foreign antigens [49].

Cancer cells can evade the immune system by secreting immunosuppressive cytokines that can downregulate major histocompatibility molecules, can recruit regulatory T-cells, and can kill reactive cytotoxic lymphocytes. Thus, the tumor microenvironment is highly immunosuppressive, which allows a tumor to grow and metastasize [77]. Several efforts have been undertaken to manipulate the tumor microenvironment in order to induce tumor regression.

## Innate immunity

Most tumor cells express antigens that can mediate antitumor immune responses [78]. Earlier studies on antigens for therapeutic targeting were based on shared antigens that are expressed on self-tissue or peripheral cells, which can lead to immunologic tolerance for the interaction between antigen peptide, major histocompatibility complex (MHC), and T-cell antigen receptor (TCR). Generated immunologic responses were restricted with low therapeutic efficacy [78]. Recently, it has been found that neoantigens generated by point mutation in normal genes, which are unique to particular tumors, can result in much more potent antitumor T-cell response. Some cancers display hundreds or even thousands of mutations in coding exons, representing a large resource of potential targets for recognition by the immune system. However, despite such a plethora of antigens, most cancers progress and evade immune-system mediated destruction [78].

Antigens recognition by dendritic cells induce a T-cell inflamed reaction consisting of infiltrating T-cells, a broad chemokine profile, and type I interferon signature indicative of innate immune activation. Presence of excessive infiltration by CD8+ cells both within the tumor and in the peritumoral stroma (high immunoscore) had a favorable prognostic significance with improved survival, even in advanced cancer stages, compared to tumors with poor or no T-cell infiltration (low immunoscore) at an earlier stage of malignancy [79–81]. Hence, a heavy presence of activated CD8+ T-cells reflects good innate immune responses against such malignancy. Recent investigations have suggested two explanations for tumor escape recognition by host immune system, based primarily on cellular and molecular characteristics of the tumor microenvironment [78].

**Figure 2:**
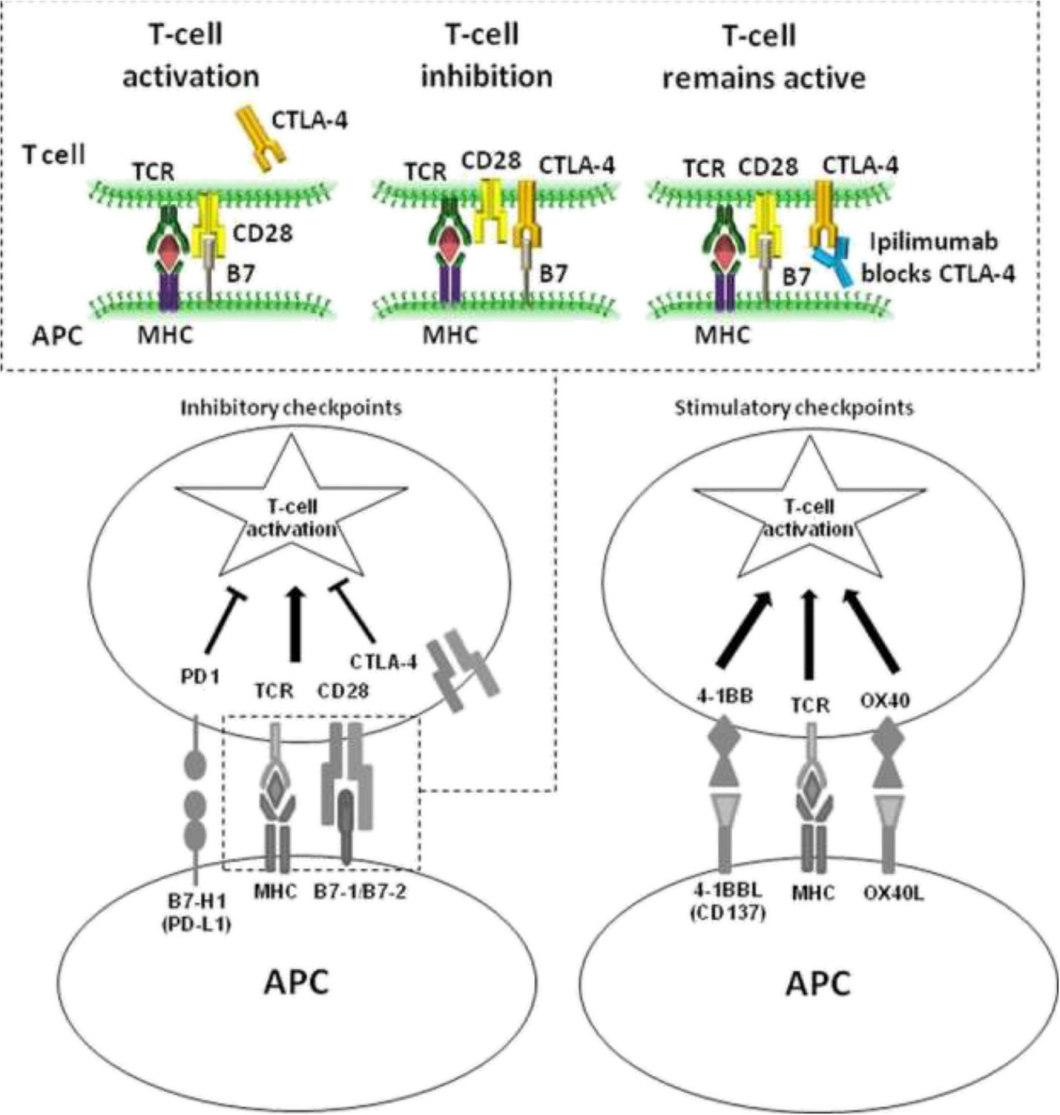
**Mechanism of action of monoclonal antibody ipilimumab.** Generation of an immune signal requires presentation of tumor antigen by major histocompatibility complex (MHC) class I or II molecules, on an antigen presenting cell (APC) such as dendritic cell. However, T-cell activation and proliferation requires a second signal, typically generated by CD28 antigen. When CD28 antigen on T-cell surface simultaneously binds to costimulatory B7-1/B7- ligand on the antigen presenting cell (APC), T-cell upregulate and translocate CTLA-4 receptor molecules to the surface, which binds B7 with a higher avidity than CD28, leading to suppressor effects, with T-cell inhibition, reduction of interleukin-2 (IL-2) secretion, and prevention of immune response against malignant cell. Ipilimumab blocks cytotoxic T-lymphocyte antigen-4 (CTLA-4) receptor, thus prevents such inhibitory effect, and allows T-cell to proliferate and mediate an immune reaction against malignant cells. Other regulatory checkpoints with the potential for modulation include the coinhibitory molecule PD-1, as well as costimulatory molecules such as OX40 and 4-1BB. Abbreviations: APC, antigen presenting cells; CTLA-4, cytotoxic T-lymphocyte antigen-4; MHC, major histocompatibility antigen; TCR, T-cell receptor. (Courtesy of Annals of New York Academy of Science and Wiley, Hoboken, New Jersey, Publisher) [86].

**Figure 3:**
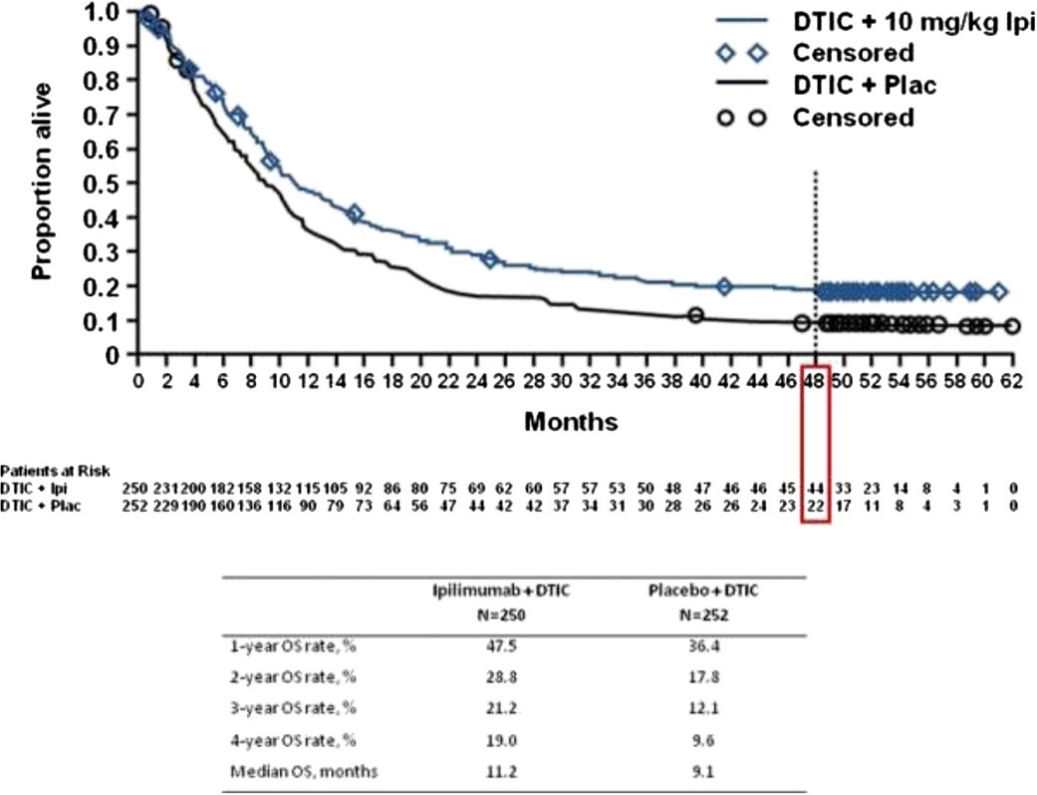
**Analysis of overall survival comparing monoclonal antibody ipilimumab plus dacarbazine to placebo plus dacarbazine in metastatic melanoma patients.** Kaplan–Meier analysis of overall survival in the phase III study CA184-024. Survival analysis of overall survival in treatment-naive patients with advanced melanoma who received ipilimumab at 10 mg/kg plus DTIC or placebo plus DTIC in the phase III trial, CA184-024. The survival curves reach a plateau beginning at approximately three years after initiation of treatment. Continued survival follow-up of more than four years demonstrates a long-term survival benefit that is consistent with the results of other ipilimumab studies. Abbreviations: DTIC, dacarbazine; Ipi, ipilimumab, Plac, placebo (Courtesy of Annals of New York Academy of Science and Wiley, Hoboken, New Jersey, Publisher) [86].

One explanation is that tumors resist immune attacks through inhibitory effects mediated by immune system suppressive pathways. This was evident in some tumors such as melanoma with high expression of PD-LI and indoleamine-2,3-dioxygenase (IDO) [82], leading to T-cell anergy and dysfunction with subsequent immune escape detection [83]. The presence of transcription factor forkhead box 03 proteins (Fox3) in the peritumoral microenvironment leads to the inhibition of tumor-infiltrating dendritic cell stimulatory functions [84]. The US FDA’s approval in 2011 of the anti-CTLA-4 monoclonal antibody ipilimumab for the treatment of patients with advanced malignant melanoma [85] represents the first-in-class strategy of uncoupling inhibitory pathways for initial antigen recognition by the host immune system [78] [Figures 2 and 3].

The other mechanism is immune system exclusion or ignorance with subsequent poor or no T-cell inflammatory reaction. Such tumors appear to lack a type I interferon signature and/or chemokines for recruitment of T-cells. Microenvironment vasculature may be nonpermissive for entry by T-cells, and the stromal element may prevent trafficking and/or function of T-cells. Radiations of tumors have shown to induce productions of interferon-beta and augment function of intratumoral dendritic cells with improved accumulation of T-cells leading to tumor regression [87]. Imatinib in gastrointestinal stromal cell tumors may cause down-modulation of IDO with improved antitumor response [88]. In patients with malignant melanoma, inhibition of R-Raf enzyme activity with vemurafenib can induce a T-cell infiltration within 1–2 weeks of therapy with some tumor responses [89]. It has been suggested that combination regimens consisting of strategies to improve innate immune system activation, T-cell trafficking in the tumor microenvironment, vaccination or adoptive T-cell transfer, and blockage of immune inhibitory pathways may be necessary to achieve clinical benefit in patients with a non-inflamed tumor phenotype. Such an approach is presently being tested in clinical trials [90, 91].

## Immunomodulatory approaches in cancer therapy

Immunotherapy in cancer can be classified into four major categories [92]. Active immunotherapy includes strategies that directly sensitize the host immune system to tumor-specific antigens, exemplified as cancer vaccines. Passive immunotherapy utilizes humanized or chimeric antibodies to specifically target tumor antigens without direct activation of the immune system. Adoptive immunotherapy utilizes patients’ immune cells, whether T-cells or dendritic cells, stimulated or manipulated ex vivo, then infused back, to better react against tumor antigens. Immune enhancement therapy aims to augment co-stimulatory molecules or block inhibitory molecules. Immune-based therapy may include one or more of the above approaches, either as distinct immunotherapy treatment, or in combination with other modalities of cancer therapy [Table 1].

### Autologous stimulated T-lymphocytes

Adoptive T-cell therapy has been shown to induce tumor regression in some patients with solid malignancies. In a recent study on patients with human papilloma virus (HPV)-induced metastatic cervical cancer who failed to respond to chemotherapy and radiation, and were selected for HPV-E6 and HPV-E7 reactivity, researchers collected tumor-infiltrating T-cell lymphocytes (TIL), and infused them back to patients. This was preceded by a non-myeloablative conditioning regimen and followed by a high-dose of bolus aldesleukin (interleukin-2). Three out of six patients with HPV reactivity achieved objective tumor responses, including two patients with metastatic disease that achieved complete tumor regression for 18 and 11 months after therapy. Side effects were minimal [93].

### Autologous activated T-lymphocytes

Host T-cell lymphocytes have been found to be successful in controlling metastatic cancer with transient side effects. The first commercially available vaccine was modified dendritic cells, sipuleucel-T (Provenge) (Dendron Corporation), which was approved by the FDA for minimally symptomatic castration-resistant metastatic prostate cancer. CD54 T-cell lymphocytes were obtained from the patients using density gradient centrifugation, and then activated ex vivo with a prostatic specific antigen in addition to granulocyte macrophage colony stimulating factor (GM-CSF) to form sipuleucel-T. Autologous activated T-cell lymphocytes, at a dose of at least fifty million CD54 cells were infused back to the patient, intravenously over 60 minutes, every two weeks for three infusions. Premedications included acetaminophen and diphenhydramine. Side effects included transient fever, chills, fatigue, asthenia, backaches, and headaches. However, infusion-induced hypersensitivity reactions with cerebrovascular events have been reported in 3.5% of patients. Compared to a control group treated with a placebo, there were significant improvements in the survival of 20.5% versus 16.1% at four years [94].

### Genetically modified activated T-lymphocytes

The adoptive transfer of lymphokine-activated lymphocytes can mediate the cellular immune response against cancer cells, which may lead to tumor regression. However, clinical trials have led to limited success. An alternative approach is to use genetically modified T-cells by altering their receptor for better recognition of tumor antigens. In such an approach, T-cells are collected from patient apheresis using density gradient centrifugation. As resting T-cell lymphocytes are non-dividing, refractory to gene therapy with lentiviral vectors, they need to be stimulated using cytokines such as interleukin-2. T-cells are then exposed to lentiviral vectors with the attached gene for 1–2 days of gene transfer. After transduction by the lentivirus, cells are then stimulated further to obtain a therapeutically effective number of cells. Genetically modified T-cell lymphocytes are then re-infused back into the patient [95]. The high-affinity of modified T-cells in detecting very low levels of tumor antigens is an extremely potent approach against tumor cells. However, it may also destroy normal cells. In another study using T-cell receptor gene-modified cells against melanoma differentiated antigens led to higher responses in patients with malignant melanoma [96]. It also destroyed normal melanocytes leading to vitiligo (skin depigmentation), uveitis, and hearing impairment [97].

### Chimeric antigen receptor integrated into T-lymphocytes

**Figure 4:**
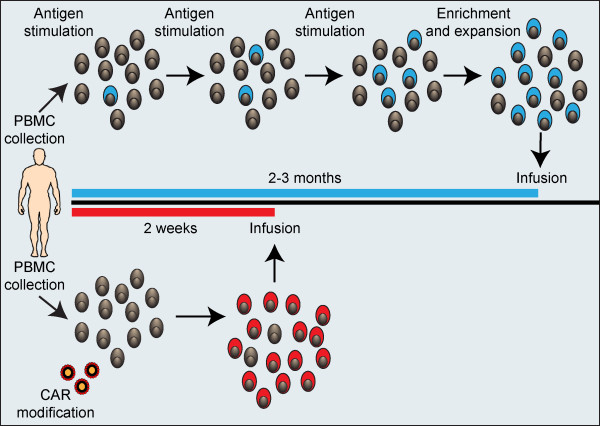
**Chimeric antigen receptor modified T-lymphocyte therapy for B-cell malignancies.** Generation of tumor-specific T cells by repeated antigen stimulation or genetic modification to express a tumor-targeting receptor. PBMC collected from a patient or healthy individual can be stimulated in vitro with tumor antigen at regular intervals to induce gradual enrichment of antigen-specific T cells (*blue*). Multiple stimulations followed by additional enrichment or expansion strategies are required to ensure sufficient antigen-specific T cells are generated. The entire process may take 2–3 months. In contrast, approaches that utilize genetic modification to redirect T cell specificity to a tumor antigen are much more rapid. PBMC can be collected from a patient or healthy donor and retrovirally or lentivirally transduced to express a tumor-reactive CAR (or TCR). The enriched CAR-modified tumor-reactive T cells (*red*) can be infused into the patient in as little as 1–2 weeks. Abbreviations: PBMC: Peripheral blood mononuclear cells, CAR: Chimeric antigen receptor modified T-lymphocytes. (Courtesy of The International Journal of Hematology, and Springer-Tokyo, Publisher) [102].

Elimination of malignant cells through host immune system depends largely on α β T-cell receptor that specifically recognize a cell target in the context of the major histocompatibility complex (MHC). Unfortunately, for many B-lineage leukemias and lymphomas, the resident immune system of patients remains incapable of controlling tumor growth, since autologous T-cells lack expression of the required receptors and tumor cells have adapted to evade immunological recognition [98]. It has been demonstrated that a chimeric antigen receptor (CAR) integrated into T-cells from patient (or even from healthy individuals), can directly recognize the CD19 molecule expressed on the cell surface of B-cell malignancies independent of major histocompatibility complex [99]. Recently, CD19-specific chimeric antigen receptor redirected T-lymphocytes have been utilized as gene therapy for patients with B-cell malignancies. One approach is to use a microelectroporator to achieve high throughput non-viral gene transfer of naked DNA plasmid, of in vitro transcribed CAR mRNA into human T cells that had been numerically expanded ex vivo using interleukin-2. After electroporation, a procedure that usually takes about 10 minutes, up to 80% of the passaged T-cells expressed the CD19-specific CAR, with redirected effector function of the genetically manipulated T-cells to specifically lyse CD19+ tumor cells. Preserved T-cells can then be re-infused into patient as an effector form of adoptive immunotherapy [98] [Figure 4]. Similar approaches have been used against other B-lineage restricted antigens such as CD20 in lymphoma, the light chain of human immunoglobulins, or CD30 expressed by Reed-Sternberg Cells in Hodgkin lymphoma [100]. Adding costimulatory endodomain within the chimeric receptors such as CD28, 4-1BB, or their combination, usually leads to enhancement of T-cell functions through the release of interleukin-2, interleukin-7 or interleukin-15 cytokines [100]. Excellent results in patients with B-cell malignancies have been reported [101–103]. CAR-modified allogeneic T-cells, such as those obtained from healthy individuals, have the potential to act as universal effector cells, which can be administered to any patient regardless of MHC type. Such universal effector cells could be used as an 'off-the-shelf' cell-mediated treatment for cancer [104, 105].

### Genetically modified dendritic cells

Dendritic cells are the most powerful antigen-presenting cells (APCs) for antigen identification, T-cell costimulation and cytokine production by T lymphocytes [106]. Dendritic cells can be generated from monocytes or a CD34+ cell precursor [107], and can be harvested from peripheral blood using density gradient centrifugation. When cultured in the presence of granulocyte-macrophage colony-stimulating factor (GM-CSF) plus interleukin-4, monocytes develop into immature dendritic cells in 3–5 days. Cells are then exposed to a variety of different stimuli over another 2 days in culture media to become mature dendritic cells, which are more effective in stimulating T-cells [108]. To target a specific tumor, mature dendritic cells are incubated with certain peptides, proteins, or irradiated tumor cells. An alternative approach is genetic modification of dendritic cells through viral and non-viral gene transfer vectors, resulting in dendritic cells that are more potent in antitumor immunity. Dendritic cell vaccines have a favorable safety profile, with toxicities limited to a local inflammatory reaction, flu-like symptoms, and vitiligo-like skin changes [109]. Despite numerous clinical trials, clinical outcomes have been modest. Emphasis has been shifted in using therapy on patients with a lower tumor burden, such as those after surgery, or following successful chemotherapy or radiation therapy. Indeed, significant synergy has been observed for chemotherapy and dendritic cell vaccines [107].

### Genetically modified tumor cell vaccine

Previous attempts to use tumor cells or their products as a vaccine have not been successful. Subsequently, clinical trials have been conducted using methods to increase tumor antigenicity in order to enhance the immune-mediated tumor lysis by T-cells. One approach is to obtain tumor cells, infect them with a viral vector such as recombinant poxvirus that contains multiple costimulatory molecules to enhance the immunogenicity of tumor cells, and subsequently use those modified tumor cells as a vaccine. An alternative approach is to directly administer the poxviral vector into the tumor. Such an approach enhances tumor antigenicity and subsequent antigen-specific T-cell response, leading to an antitumor effect. Other viral vectors include vaccinia virus and recombinant nonreplicating fowlpox virus encoding GM-CSF (TRICOM) [110]. This has led to the development of the Prostvac-VF vaccine (Bavarian Nordic, Inc.) which employs a recombinant vaccinia vector as a primary vaccination, followed by multiple booster vaccinations employing a recombinant fowlpox vector. Both vectors contain the transgenes for prostate-specific antigen (PSA) and multiple T-cell costimulatory molecules (TRICOM). In a randomized clinical study, using the vaccine versus placebo, in 125 patients with metastatic castration resistant prostate cancer, there was an improved survival at 3 years of 30% for the vaccinated group compared to 17% for the placebo patients [18]. A Phase-III clinical trial is presently in progress.

### Single-antigen plasmid-based vaccine

DNA plasmids containing a genetic sequence that encodes a desired antigen with other transcriptional elements have been considered as a mode of cancer vaccine. It readily accesses the nucleus of a transfected cell, transcribed into a peptide or protein, and may lead to cellular and humoral immune response [111]. The technique is considered to be relatively safe compared to viral or bacterial vectors, does not cause infection or autoimmune disorders, and is easy to develop and produce commercially [112]. However, its effectiveness wanes with time. Hence, the need for frequent booster immunizations. Examples of single-antigen plasmid-based vaccines include human prostatic acid phosphatase protein for patients with prostate cancer [113], human epidermal growth factor receptor-2 (HER-2/neu), protooncogene with low-doses of GM-CSF intradermally for patients with metastatic breast cancer [114], and modified carcinoembryonic antigen (CEA) gene fused to a promiscuous tetanus toxoid for colorectal cancer [115]. Although therapy was well tolerated, responses were minimal and transient. Using a multiple-antigens plasmid-based vaccine leads to broadly specific, long lasting, and multifunctional immune stimulation [116]. Improved results were noticed [117, 118].

### Genetically modified microenvironment

The microenvironment around a tumor plays an important role in tumor progression and metastases. It includes stromal tissue, fibroblasts, and vascular endothelial cells. Interfering with such a microenvironment will lead to tumor regression. The most important target is angiogenesis, which is essential for tumor growth and metastases. It is mediated by tumor-derived pro-angiogenic cytokines, such as the vascular endothelial growth factor and fibroblast growth factor. These factors stimulate the proliferation of microvasculature around a tumor, with subsequent tumor progression and metastases. Compared to the recombinant antivascular endothelial factor antibody “bevacizumab”, gene therapy represents an attractive alternative to such drug therapy. Using an anti-angiogenic genes, such as *angiostatin* and *endostatin*, delivered by an adeno-associated virus vector, has led to tumor regression with minimal side effects [24].

## Other gene therapy approaches in cancer management

As with other modes of cancer therapies, multimodality treatment frequently yields, better results compared to monotherapy. This is similarly true for gene therapy, and is evident when gene therapy is administered after maximum tumor load reduction following radical surgery or successful chemotherapy. Gene therapy has a synergistic effect when combined with chemotherapy, with higher tumor responses and lower therapy-related toxicities.

### Gene directed enzyme prodrug therapy (GDEPT)

This is a new strategy in cancer management that aims to reduce the side effects of chemotherapy. With such an approach, a gene that expresses a nontoxic enzyme into cancer cells is first delivered to the cells, followed by the systemic administration of a pro-drug that can be converted into a toxic compound by the enzyme, leading to selective tumor cell death, with lower adverse effects on normal tissues [119]. Cell-to-cell diffusion of toxic metabolites may damage nearby and adjacent tumor cells (bystander effect) [120]. Release of tumor cell necrotic material in the circulation may activate the immune system in response to the tumor antigen, with subsequent regression of distant tumor cells, such as metastatic nodules (distant bystander effect) [121]. Examples include the use of a retroviral vector, such as suicide gene therapy and herpes simplex virus carrying the thymidine kinase enzyme, to the interior of tumor cells. The enzyme has a 1000-fold greater efficiency to selectively phosphorylate the acyclovir-derived pro-drug ganciclovir [120]. Following the systemic administration of ganciclovir, the drug is metabolized in tumor cells leading to cell death. As the efficacy of such a system is only about 10% of tumor cells, the extent of tumor regression is mainly mediated via bystander effects. The system has been tried in several clinical trials [122]. Replacing ganciclovir with a penciclovir drug, modified to generate radiolabeled analog, will also allow a closer follow-up of therapy results, using high-quality positron emission tomography imaging studies [123].

### Cancer drug-resistance gene transfer

Several studies have used a gene transfer approach that aims to enhance chemotherapy and radiation effects against cancer cells, while protecting normal tissue against therapy mediated toxicities. Such gene transfer may also be used in the protection against HIV virus by making normal cells resistant to viral invasion, or correction of genetic disorders such as sickle cell anemia or metabolic disorders. However, incorporating a new gene into a host stem cell’s genome, for the life of an individual, may promote other oncogenes to develop malignant disorders, and may change other adjacent genes, thus creating other medical diseases. Hence, it is a risky approach in gene therapy. Few clinical trials have recently been conducted in this regards. One example is the multidrug-resistant protein-1, which is encoded by the human *ABCBI* gene named as *MDR1* gene. It stimulates the cellular pump to remove cytotoxic drugs from normal cell cytoplasm to the outside, thus protecting normal cells from chemotherapy’s side effects, such as with vinca alkaloids, taxanes, epipodophyllotoxins and anthracyclines [124]. The *MDR1* gene is minimally expressed in malignant cells; thus, chemotherapeutic medications entering the cytoplasm will remain at a higher concentration, leading to cell death. Other drug-resistant genes include methyl guanine methyltransferase (*MGMT*) for alkylating chemotherapy [125, 126], and glutathione transferase (*GSTP1*) for cisplatin, doxorubicin, and cyclophosphamide [127, 128, 124].

### Theranostic approach

In a combined diagnostic and therapeutic system (theranostic), gene therapy may also be combined with other diagnostic measures to help diagnose, treat and monitor the response to therapy. For example, a small interfering double-stranded RNA (siRNA) delivery system can be labelled with imaging agents such as dextran-coated superparamagnetic nanoparticles for simultaneous noninvasive imaging of siRNA delivery to tumors, using magnetic resonance imaging (MRI) [59]. The siRNA delivery system can also be labeled with other imaging agents to closely monitor therapy, and may even predict the outcome of therapy long before any anatomical changes [129]. Such molecular diagnostic approaches have been evolving relatively fast in the last few years, and may become an important avenue in cancer diagnosis sometime in the near future [59].

## Problems with gene therapy

The most frequent side effects following gene therapy include transient fever and flu-like symptoms [24]. A grade-3 hypersensitivity reaction following intravenous administration is usually transient and managed with the usual supportive measures. Leukocytopenia, and in particular, lymphopenia, may represent cellular redistribution of white blood cells to target tissue such as tumors. Mild transient anemia has also been reported [130]. However, toxicity, mutagenicity and immunogenicity associated with viral vector therapy have raised great concern [12].

Retroviral (such as lentiviruses) mediated gene therapy leads to viral integration into host genome, thus, it may cause mutagenic events with possible second malignancies. This was reported in earlier studies on the murine leukemia retrovirus vector in the treatment of patients with severe combined immunodeficiency and five out of 30 cases developed leukemia [131], though, no second malignancy has been reported so far, in gene therapy for cancer. Such mutagenicity depends on the site of viral insertion. For this reason, the FDA has required all clinical trials involving genomic integrated viral vectors to report and analyzes viral vector insertion sites. Initial methodology was linear amplification mediated polymerase chain reaction [132], but lately, high-throughput DNA sequencing methods have been used [133, 134]. Clinical trials that initially or subsequently show evidence of higher mutagenicity are usually discontinued. Information obtained from such studies is of major significance in designing new and much safer therapeutic approaches [58].

Another major problem with gene therapy for cancer is the resistance to treatment with subsequent tumor recurrences and shorter survival. A potential mechanism is intrinsic, and possibly acquired, tumor cell resistance to therapy-induced cell death (apoptosis) by dysregulation and release of anti-apoptotic inhibitor of apoptosis protein or Bcl-2 proteins [24]. Recently, some pharmaceutical companies have developed several medications such as Novartis-LBH589, cIAP1, and cIAP2 which inhibit the Bcl-2 protein, thus promoting cell death (apoptosis) and tumor regression, prevent or delay tumor resistance, and prolong remission following gene therapy. These medications are presently in clinical trials [24, 135].

## Review, Conclusions

Gene therapy for cancer has evolved relatively fast in the last two decades, and presently, few drugs are commercially available while others are still in clinical trials. Most reports on gene therapy have shown good safety profiles with transient tolerable toxicities. The lack of success in several clinical trials may partly be attributed to patient selection. Similar to initial chemotherapy outcomes thirty years ago, patients with advanced and therapy-resistant malignancies are presently enrolled in gene therapy trials. Perhaps, gene therapy maybe much more successful in patients with earlier stages of malignancies, or in those who have a lower tumor burden. Alternatively, gene therapy may better be used after successful cancer therapy with maximum tumor load reduction, such as after radical surgery, following radiation therapy, or after successful chemotherapy.

In the future, the wide use of patient and tumor genomic analysis as well as the assessment of host humoral and cellular immunity, will facilitate a better selection of the most appropriate gene therapy per patient. Recent progress in developing safe and effective vectors for gene transfer, such as with synthetic viruses and non-viral methods, as well as the success in using autologous and allogenic chimeric antigen receptor integrated T-lymphocytes, even from healthy individuals, as universal effector cells in mediating adoptive immunotherapy, will increase the effectiveness and safety profile of gene therapy. Furthermore, with the advancement in biological research, much cheaper gene vectors will become commercially available, which will make gene therapy readily available to the majority of cancer patients, worldwide. This will transform the future of cancer therapy, from generalized cancer treatment strategies, based on tumor size, nature and location, to a more tailored, individualized cancer therapy, based on the patient’s specific genomic constituents, host immune status, and genetic profile of the underlying malignancy. Treatment is expected to be fast, effective, relatively less toxic and inexpensive, with higher cure rates, and may even, cancer prevention.

## Authors’ information

The author is an internist and medical oncologist at a regional cancer center in the United States, with higher qualification from United Kingdom, Canada, and United States, previous academic title at Ohio State University, over thirty five years practice as a medical oncologist, and authored or co-authored over twenty five peer-reviewed papers on cancer management and research.

## Abbreviations

APCs: Antigen-presenting cells
CEA: Carcinoembryonic antigen
DNA: Deoxyribonucleic acid
DTA-H19: Corynebacterium diphtheriae toxin-A chain
EGFR: Epidermal growth factor receptor
Fox3: Forkhead box 03 transcription factor protein
FDA: The Food and Drug Administration in the United States
GDEPT: Gene directed enzyme prodrug therapy
GM-CSF: Granulocyte macrophage colony stimulating factor
HER-2/neu: Human epidermal growth factor receptor-2
HIV: Human immunodeficiency virus infection
hTERT: Human telomerase reverse transcriptase
IDO: Indoleamine-2,3-dioxygenase
MHC: Major histocompatibility complex
MDR1: Multidrug-resistant protein-1
mRNA: Messenger-RNA
NK: Natural killer cell lymphocytes
PSA: Prostatic acid phosphatase antigen
RISC: Ribonucleic acid induced silencing complex
RNA: Ribonucleic acid
RNAi: Ribonucleic acid interference
siRNA: Small interfering double-stranded RNA
TAAs: Tumor-associated antigens
TCR: T cell antigen receptor
TRICOM: Multiple T-cell costimulatory molecules
VEGF-A: Vascular endothelial growth factor A receptor.
